# Quantum Crystallography as a Chemist's Tool for Bond Analysis

**DOI:** 10.1063/4.0000803

**Published:** 2025-10-27

**Authors:** Floran Meurer, Michael Bodensteiner

**Affiliations:** University of Regensburg, Regensburg, Bavaria, Germany

## Abstract

The insights from the quantum crystallographic analysis of three different cases are highlighted. Two are based on the combined methods of an “experimental charge density” coming from a multipolar model (MM) [1], and an “experimental wavefunction” coming from X-ray restrained wavefunction (XRW) fitting [2]. In both examples, the effects of different wavelengths for the data collection and the refinement of anomalous dispersion parameters are highlighted.

In the first example (Fig. 1) the ylid/ylene and carbonyl/enolate equilibrium in WYLID is investigated as a contribution to a long ongoing debate in the quantum crystallography community about this system. This compound is of particular interest as it closely resembles YLID, the most measured single-crystal structure in the world. [5,6] Complementary bond analysis from both, the MM and XRW shows that the carbonyl form is the most suitable description for the bonding situation in WYLID and YLID (see Fig. 2).

The second example shows how the reactivity of the organometallic synthon for white phosphorus [Ni]cyclo-P3 ([Ni] = 1,2,3-trimethyl- cycplopentadienylnickel) can be explained and categorized as a metallatetrahedrane using MM and XRW. [3] Experimental frontier orbitals from the XRW fitting were able to correctly capture the reactivity of [Ni]cyclo-P3 which was not able by previous studies. [4] Additionally, the first multipolar model and XRW using Cu Kβ was part of this study.

The third case highlights the potential of Quantum Crystallography to verify or disproof chemical theoretical calculations. In the case of a Th3 cluster [7], we show how the deformation density provides valuable information on the agreement of the calculated electron density coming from density functional theory and the experimental electron density from a single-crystal X-ray diffraction experiment (Fig. 3).
